# Pulmonary artery sarcoma affecting the pulmonary valve mistaken as pulmonary vasculitis: a case report and comparative literature review

**DOI:** 10.1186/s13019-024-02700-3

**Published:** 2024-05-14

**Authors:** Harry James Smith, Fadi Al-Zubaidi, Daniel Sitaranjan, Steven Chiu, David Jenkins

**Affiliations:** Royal Papworth Hospital, Papworth Road, Trumpington, Cambridge, CB2 0AY UK

**Keywords:** Pulmonary artery sarcoma, PAS, Pulmonary thromboendarterectomy, Pulmonary valve replacement, Pulmonary vasculitis

## Abstract

Pulmonary arterial sarcomas (PAS) are rare aggressive tumours occurring mainly in the pulmonary trunk. We report a case of PAS involving the pulmonary trunk wall and valve, with uniform wall thickening which represents an atypical imaging manifestation of this tumour. A 63-year-old male presented with vague respiratory symptoms with rapid progression. CTPA showed low density filling defects in both pulmonary arteries and PET scan showed increased uptake in the pulmonary trunk, which along with raised ESR suggested Pulmonary Vasculitis. Echo imaging showed Right ventricular hypertrophy and pulmonary stenosis. Response to steroid therapy was minimal and his symptoms worsened. A referral for second opinion was made and he was diagnosed with PAS. He underwent Pulmonary thromboendarterectomy with Pulmonary valve replacement. Post-operative histopathology confirmed the diagnosis. PAS is rare and frequently misdiagnosed. Surgical resection is not curative, but together with chemotherapy can prolong survival.

## Introduction

Pulmonary artery sarcoma with pulmonary valve involvement is rare(Bandyopadhyay et al. 2016). Patients with PAS often report vague symptoms such as dyspnoea, haemoptysis, chest pain, cough, and fever[11]. The listed symptoms are not necessarily specific to PAS and hence misdiagnosis is frequent[14].

Involvement of the pulmonary valve in PAS is unusual and infrequently reported[8]. Pulmonary valve replacement in adults is also rare and often because of congenital cardiac condition management[12]. The main goal for surgical intervention is to remove obstructive tissue and to reduce pulmonary vascular resistance and reverse RV remodelling. Common markers of RV function used in the improvement are end-systolic volume (< 82 ml/m2 to 90 ml/m2) and RV ejection fraction (whereby EF of less than 30% is associated with major adverse clinical outcomes)[12, 5].

Patients diagnosed with PAS often have poor survival and in cases in which the tumour is unresectable or already metastasised [4, 13]. In certain cases, median survival has been shown to be 45 days [2]. However, if treated with chemotherapy the median survival ranges from 8 to 17 months [6]. The use of chemotherapy/radiotherapy and surgery together shows prolonged median survival of 26 months [3, 2]. Without surgical treatment the average duration of life of PAS patients from diagnosis is approximately 1.5 months [14].

## Case presentation

A 63-year-old retired male was referred to our Cardiac surgery centre reporting a history of tiredness, exertional, breathlessness and chest tightness (Class II New York Heart Association limitation). His past medical history included pre-diabetes, benign prostatic hyperplasia, and TIA with no focal neurological deficit.

The initial imaging revealed thickening in the proximal main pulmonary artery which was avid on PET. This extended into the right and left pulmonary arteries. CTPA imaging revealed a large filling defect in the main pulmonary trunk that extended into the left and right main pulmonary arteries. The main pulmonary artery was concentrically narrow and there was no infiltration through the vessel wall. Magnetic resonance imaging was concordant with CT and showed late gadolinium enhancement – these findings, particularly the avidity of uptake on PET scanning suggested a differential of large vessel vasculitis or PA Sarcoma rather than thromboembolic disease.

A transthoracic echo revealed severe pulmonary stenosis (PV max 4.67 m/s PV max PG 87mmHG) and an echogenic area around the pulmonary valve. The RV was shown to be moderately impaired and hypertrophied with estimated PA systolic pressure of 61mmHg. The left ventricle appeared preserved in size and function with an ejection fraction of 60–65%.

The patient was also staged with magnetic resonance imaging (MRI) of the head and a PET scan which were negative for distant metastasis but were suggestive of large vessel vasculitis in the pulmonary artery. The patient also had an elevated ESR 40 mm/h, this guided initial high dose steroid treatment.

**Fig. 1:**
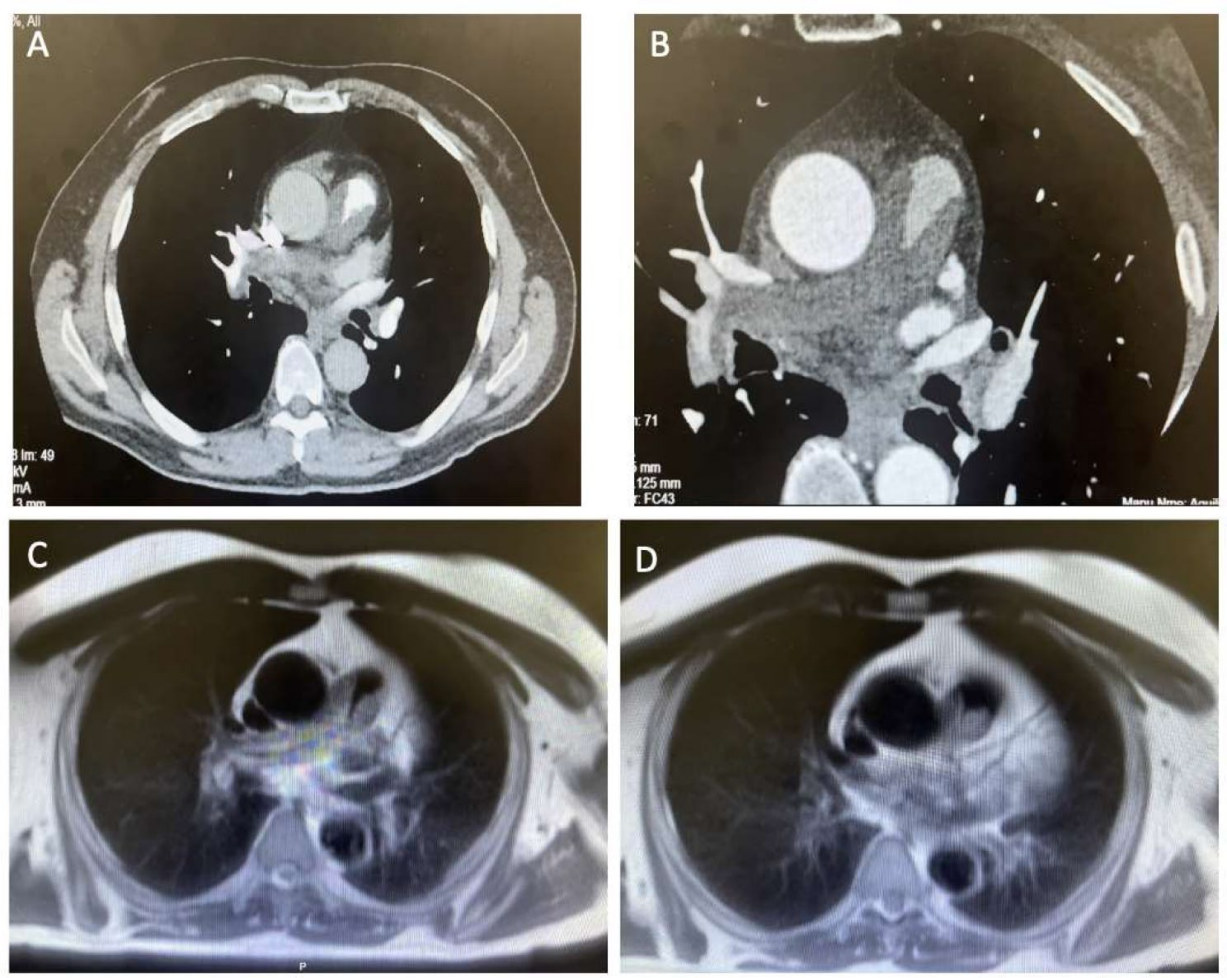
Images **A** and **B** show pre-operative CTPA and images C and D show pre-operative cardiac MRI

The patient responded initially to high dose steroid therapy– this led to a brief improvement of symptoms and reduction in ESR to 25 mm/h. He was re-imaged and there appeared to be no radiological improvement of the defects and his symptoms began to progress (Fig. 1). He eventually was referred to our centre for further investigation.

The suspicion of pulmonary artery sarcoma (PAS) increased, and he was discussed at the Pulmonary Vascular Disease MDT. Biopsy of intra pulmonary artery masses is not usually recommended, but a biopsy was attempted at his referring centre, but this was unsuccessful due to pain and haemodynamic instability.

Collectively the decision was made for pulmonary thromboendarterectomy and pulmonary valve replacement. Upon attendance to our centre the patient revealed his symptoms had progressed over the 2-month period since his initial investigations from New York Heart Association (NYHA) class II to class III.

**Fig. 2:**
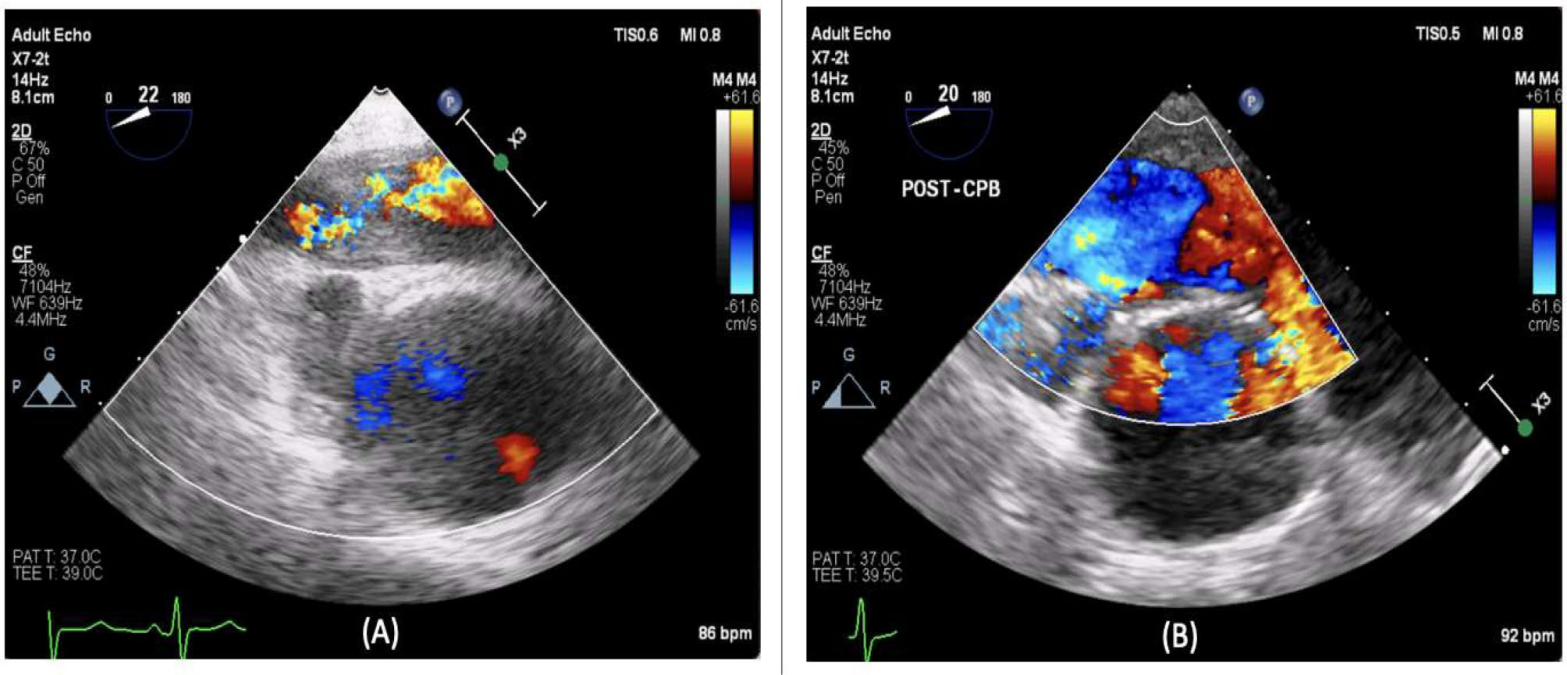
Shows a transoesophageal (Mid oesophageal, short axis, ascending aorta, colour flow doppler) (**a**) is preclearance and (**b**) is post clearance

**Fig. 3:**
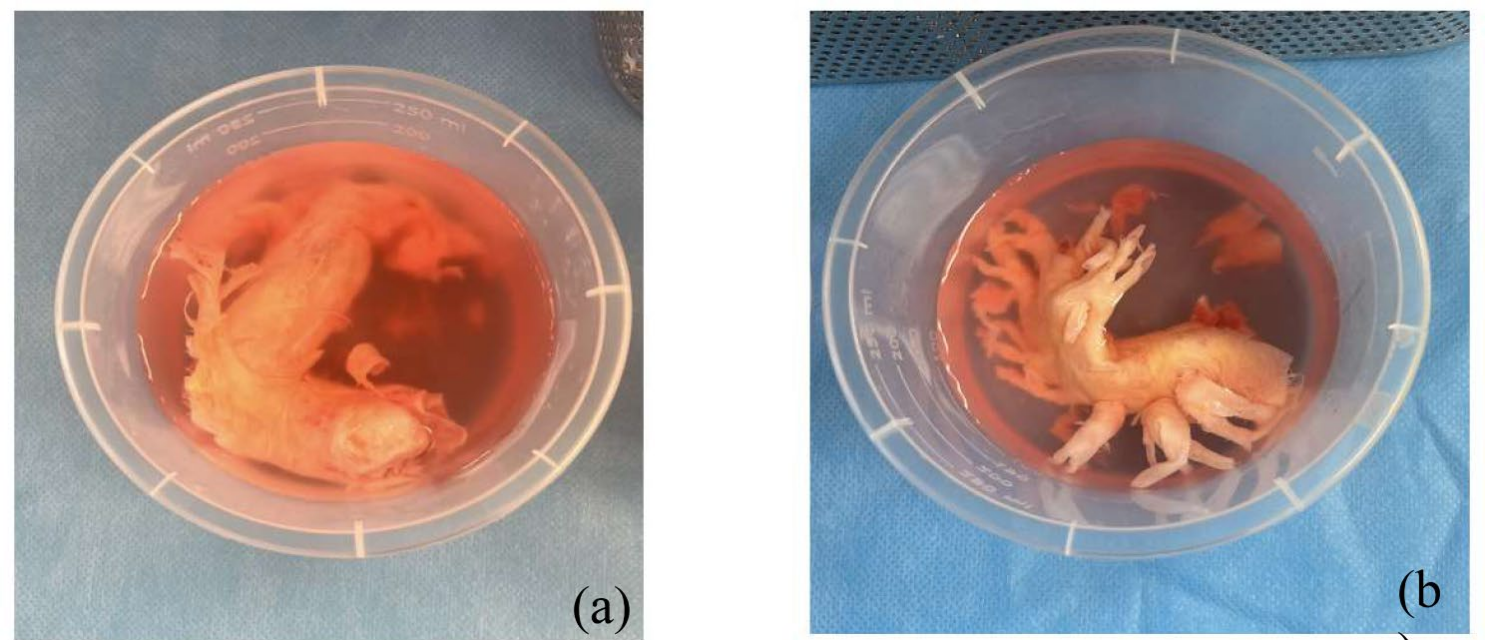
shows the histology samples that were taken from the right and left pulmonary trunks (**a**) shows the left pulmonary artery cast including stenosed pulmonary valve (**b**) shows the right main pulmonary artery cast

**Fig. 4:**
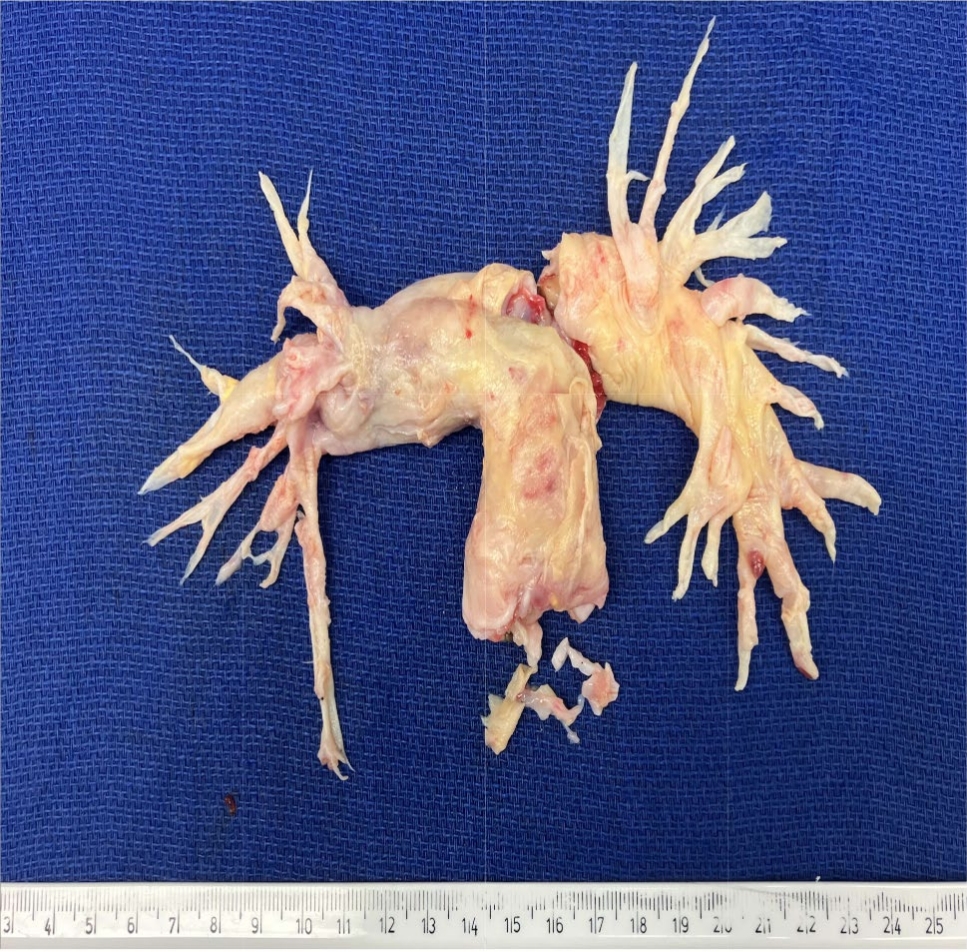
The removed pulmonary artery sarcoma with scale reference in centimetres

The surgery was performed on cardiopulmonary bypass under deep hypothermic circulatory arrest at 20 degrees Celsius as standard for pulmonary endarterectomy. Both pulmonary arteries were opened simultaneously for access under the aorta to facilitate the endarterectomy and removal of the sarcoma. Excellent clearance was achieved of upper lobe, middle lobe, and lower lobe of the right and left main pulmonary artery (Figs. 2, 3 and 4). The pulmonary valve was tri-leaflet and was excised and replaced with a 25 mm Perimount magna bioprosthesis. The pulmonary artery was closed with a bovine pericardial patch. Total bypass time was 301 min with a total deep hypothermic circulatory arrest time of 16 min, cross clamp time was 102 min.

Pulmonary artery sarcoma was confirmed by histopathology services. Histopathology results from the right and left pulmonary artery casts showed both specimens to have extensive variable cellular intimal thickening by tumour composed of spindle and cuboidal cells with some accentuation beneath the luminal surface. There was a surrounding vascularised fibrous stroma. The cells showed weak patchy staining for actin and CD31 but not CD34 or desmin. The appearances are in keeping with an intimal sarcoma. The pulmonary valve had focal involvement of the valve by tumour cells.

After this diagnosis the patient was referred to medical oncology for discussion of further adjuvant chemotherapy. The patient has been reviewed at follow up and has a self-reported improved quality of life and remains alive 10 months from surgery.

## Discussion

This report highlights some of the difficulties in the initial diagnosis of PAS, often confused with more common thrombus, but unusually in this case it was initially diagnosed vasculitis. The ineffective nature of the vasculitis treatment combined with the progression of his symptoms suggested an alternative diagnosis. It is important to consider PAS as a diagnosis in patients with rapidly progressing symptoms and an increasing intra pulmonary artery mass on CT.

Vasculitis is an autoimmune inflammation of blood vessels resulting in constitutional symptoms and organ damage [9]. Most pulmonary manifestations of vasculitis are non-specific, and the lung is primarily targeted by small vessel vasculitis, but large and medium vessel vasculitis has been reported in the literature [15].

The recommended diagnostic work ups for PAS and vasculitis are similar (CTPA, MRI, PET imaging) [15]. Comparing Pulmonary artery Sarcoma and Pulmonary artery vasculitis reveal several important distinguishing factors.

Vessel wall thickening and aneurysmal dilation of the pulmonary vessels are radiological features of vasculitis seen on MRI, PET and CTPA.[15]. In our case report, wall thickening was seen on MRI and PET but there was no aneurysmal dilation of the pulmonary vasculature. Distension by the mass on imaging studies and compression of neighbouring structures are clues to a diagnosis of PAS [16]. MRI imaging has also been shown to be useful in diagnosis when distinguishing tissue from pulmonary embolism thrombus [7].

Heterogenous densities, centrally located with large filling defects are typical of PAS and often represent haemorrhage, necrosis, and ossification [16]. These points must be considered when considering a diagnosis of pulmonary thromboembolism or pulmonary vasculitis to reduce the risk of delayed diagnosis and therefore delayed treatment.

Vasculitis leading to pulmonary valve stenosis is not reported in the literature. In contrast, pulmonary artery sarcoma has been shown to involve the pulmonary valve and right ventricular outflow tract in some cases [11, 2]. Therefore, the use of TTE and TOE imaging is useful in distinguishing between vasculitis and PAS and should be performed to assist with operative planning.

Generally, patients with vasculitis are treated medically to suppress inflammation. Very occasionally surgery may be possible to relieve short segments of residual proximal stenosis in the pulmonary artery following quiescence of the disease. The initial post operative management of these patients is similar providing the vasculitis has been adequately resected and any inflammatory episodes are appropriately managed. One case report in the literature reports a patient undergoing pulmonary thromboendarterectomy for suspected CTEPH but who, in fact, had been shown on autopsy to have giant cell arteritis [3]. This patient had a poor outcome, and it highlights the need for accurate diagnosis to best manage the patient pre-operatively in terms of their work up for surgery and post-operatively in terms of their disease-free survival. Pulmonary hypertension can exist because of PAS with concomitant chronic thromboembolism, all of which can manifest as vague respiratory symptoms [10]. This highlights the value of multimodality imaging when pulmonary artery sarcoma is suspected.

Despite the similarities between PAS and vasculitis our case highlights some of the cardinal features of malignancy that are not seen in large vessel vasculitis. No formal guidelines currently exist for the management of this condition.

## Data Availability

Data and material is available by request to first author.
